# Minimizing the COVID-19 spread in hospitals through optimization of ventilation systems

**DOI:** 10.1063/5.0081291

**Published:** 2022-03-02

**Authors:** Hamed Arjmandi, Reza Amini, Mehdi Kashfi, Matthew Alexander Abikenari, Ashkan Davani

**Affiliations:** 1 MR CFD LLC. No 49, Gakhokidze Street, Isani-Samgori District, Tbilisi 0182, Georgia; 2 Department of Orthopedic Surgery Research Center, University of California Los Angeles, Los Angeles, California 90095, USA; 3 Department of Aerospace and Mechanical Engineering, University of Southern California, Los Angeles, California 90007, USA

## Abstract

The rapid spread of SARS-CoV-2 virus has overwhelmed hospitals with patients in need of intensive care, which is often limited in capacity and is generally reserved for patients with critical conditions. This has led to higher chances of infection being spread to non-COVID-19 patients and healthcare workers and an overall increased probability of cross contamination. The effects of design parameters on the performance of ventilation systems to control the spread of airborne particles in intensive care units are studied numerically. Four different cases are considered, and the spread of particles is studied. Two new criteria for the ventilation system—viz., dimensionless timescale and extraction timescale—are introduced and their performances are compared. Furthermore, an optimization process is performed to understand the effects of design variables (inlet width, velocity, and temperature) on the thermal comfort conditions (predicted mean vote, percentage of people dissatisfied, and air change effectiveness) according to suggested standard values and the relations for calculating these parameters based on the design variables are proposed. Desirability functions that are comprised of all three thermal condition parameters are used to determine the range of variables that result in thermally comfortable conditions and a maximum desirability of 0.865 is obtained. The results show that a poorly designed ventilation system acts like a perfectly stirred reactor—which enormously increases the possibilities of contamination—and that when air is injected from the ceiling and extracted from behind the patient beds, the infection spread is least probable since the particles exit the room orders of magnitude faster.

## I. INTRODUCTION

The new COVID-19 virus pandemic has inflicted tremendous damage upon the world. In only one year since its emergence in December 2019, more than 100 million people have been diagnosed with the virus infection globally, which has led to more than 2 × 10^6^ deaths.^1^ SARS-CoV-2 is a severe acute respiratory syndrome that causes COVID-19 and has a higher fatality rate in elderly patients and those with preexisting pulmonary conditions.^2^ Although initially it was anticipated that the SARS-CoV-2 virus is transmitted through droplets from sneeze or cough (like SARS-CoV-1) and not by airborne particles and that social distancing may help break the infection chain,^3^ new studies suggest that the virus can indeed be transported through the air.^4^ The rapid spread of virus has overwhelmed hospitals with patients in need of intensive care, which is often limited in capacity and is generally reserved for patients with critical conditions. This has led to higher chances of infection spread to non-COVID-19 patients and healthcare workers and an overall increased probability of cross contamination. Studying the contamination spread in intensive care units is, hence, a critical step in avoiding and controlling infective diseases.^5,6^

Sahu *et al.*^7^ studied the effects of airflow blowing angle on the stability of virus-laden particles in an ICU room in eight different cases (from 10° to 80°) numerically and found that when the airflow entered the room at angles between 30° and 50°, the time for air particles to exit through air conditioning was decreased and the particle residence time was increased for angles from 70° to 80°. Ching *et al.*^8^ investigated the effectiveness of hospital curtains on airborne transmitted infections numerically and experimentally and found that curtains between beds in fact reduce their spread and are most effective when they are fully open. Verma *et al.*^5^ used CFD (Computational Fluid Dynamics) techniques to study the particle distribution evolution in a hospital room with only one bed. The authors showed how the infection spreads and highlighted the contamination paths and concluded that higher air change rates (ACRs) cause faster removal of airborne infections and emphasized that the distance to outlet is a key parameter in controlling infections. Yuen *et al.*^9^ used a suction fan in an isolation room and found that airborne particles' lifetime can significantly be reduced due to the lower local mean age of air. Cho^10^ experimentally investigated contamination dissipation in an isolation room using various ventilation strategies and concluded that the location of supply air diffusers and outlet vents greatly affects infection spread caused by breathing in the room. The author suggests that installing the HVAC (Heating, Ventilation, and Air Conditioning) vents at lower heights results in less airborne particle dissipation.

There have been numerous studies about airborne transmitted diseases in operation and isolation rooms and processes in which the infecting agent travels from the contagious patient to others in hospitals.^11–18^ Sun and Zhai presented a predicting model for airborne virus infection for indoor spaces by examining the effects of two prominent factors, viz., social distancing probability and ventilation effectiveness, and implementing them into the Wells–Riley model.^19^ Ge *et al.* investigated the exposure risk of COVID-19 virus in different rooms and sections of hospitals by collecting and testing six different air and surface samples from three hospitals for SARS-CoV-2. Their results showed that 82.6% of the samples taken from ICUs were positive and contained the DNA of SARS-CoV-2.^20^ In another study, Jin *et al.* demonstrated that air samples taken from an ICU room with only one patient who was in the recovery period from COVID tested positive even days after the patient's COVID test was negative.^21^ These reports indicate the high risk of exposure to the virus in ICUs and the need for designing an optimal air conditioning system not only for high-risk areas of hospitals, but also future indoor environments.^22^ Verma and Sinha^23^ investigated flow and temperature distributions in an ICU and showed that the stagnant regions between doctors and patients are potential contamination zones with increased chance of virus transmission. They also concluded that the arrangement and layout of patient beds are among the most critical parameters in infection control and should be considered carefully. Yu *et al.*^24^ inspected several air conditioning schemes in an ICU with focus on the internal air quality (IAQ) and found that placing the air supply unit on top and exit vent at the bottom of rooms provides better IAQ performance. They also did not find any enhancement in the IAQ by adding more air supply units, and the number and size of inlets were found to depend on the dimensions of the room. The authors also conclude that increasing the air change per hour (ACH) and decreasing the inlet velocity have considerable effects on the performance of ventilation systems. Yang^25^ studied the quality of breathing air in a four-bed hospital room with mixing and displacement ventilation methods and reported that the latter technique, which requires more air supply, provides better infection control and increases the quality of breathed air since it reduces the age of air in the room and, thus, should be the preferred method of ventilation in hospital rooms.

Although the body of work regarding the study of airborne infection spread is substantial in the literature, a majority of the investigations were carried out for isolated parameters, like particle tracking, stability and life, interactions with the flow field, performance of HVAC systems, and thermal comfort, separately and there is a lack of in-depth comprehensive studies with interactive parameters in particular, as the COVID-19 virus continues to wreak havoc on the world and the number of infected patients grows beyond hospital capacities and rapid makeshift hospitals are being built. With this motivation, the current study aims to perform a numerical modeling of infection control in a five-bed hospital room that includes the aforementioned parameters and their interactions to address challenges in inhibiting risks of airborne infection and to provide strategies that mitigate unwanted hazards associated with ventilation systems. To achieve these objectives, air flow patterns are first studied, and virus-laden particles are tracked in the presence of four different ventilation strategies. Once the best system with minimal chance of infection spread is determined, an optimization process will be carried out using the response surface method (RSM) to decide on parameters with the most influence on observables such as thermal comfort, particle residence time, and air change effectiveness (ACE). The studied parameters are the air supply channel width, inlet velocity, and temperature and the results are presented in the form of four different relations that are commonly used in HVAC analyses: predicted mean vote (PMV), percentage of people dissatisfied (PPD), residence time (RT), and ACE.

## II. METHODS

### A. Physical and numerical model

**FIG. 1.**
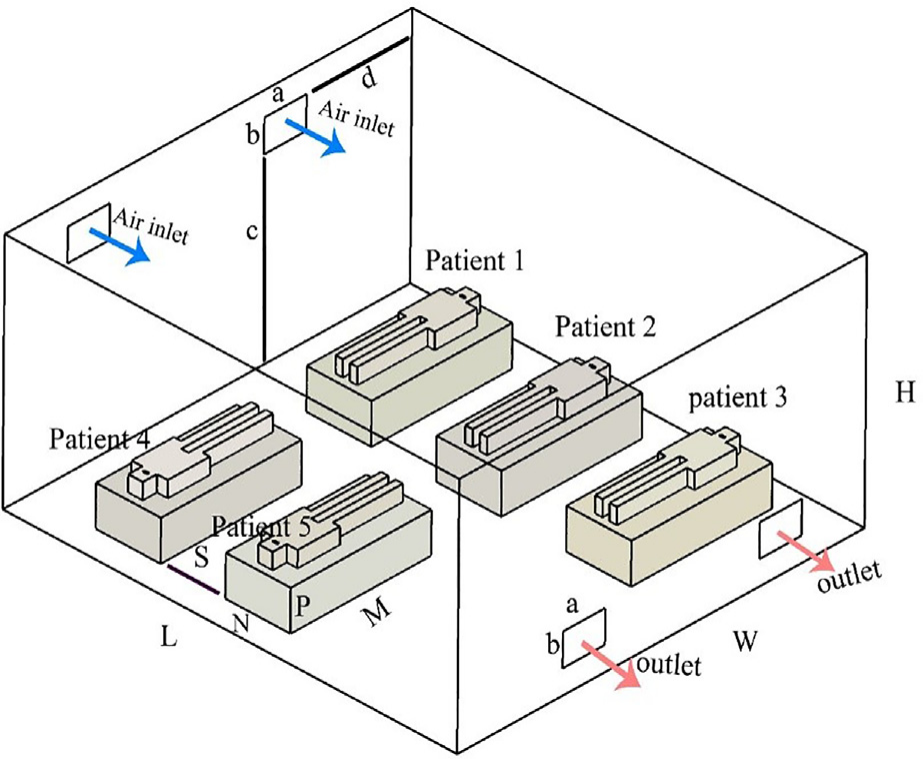
Schematic of the computational domain in the current study.

The geometry of the computational domain of the ICU room and the layout of the five patient beds in this study are shown in Fig. 1. There are two inlet and two outlet vents, which are subject to change. The dimensions of the room as well as other geometrical parameters are obtained from the work of Sahu *et al.*^7^ and are shown in Table I. The feasibility study is carried out using Reynolds-averaged Navier–Stokes (RANS) approach with the k-ε model for turbulence transport (k is the turbulent kinetic energy and ε is the turbulent dissipation); even though this is considered a second order closure model and is less accurate than higher fidelity models like Large Eddy Simulation, it requires much less computational resources and provides adequate accuracy for the current study following the validation process. ANSYS FLUENT, a finite volume computational fluid dynamics software, is used to solve the RANS-k-ε equations. The semi-implicit pressure linked equation (SIMPLE) scheme with second order upwind spatial discretization and first order implicit time discretization for transient simulations was used to solve the Navier–Stokes equations. Particles are treated as inert with one-way interaction, which means the only effective forces influencing the trajectory of particles are the primary fluid (air) momentum and the gravity. Particle injections are performed for each patient separately. Air is injected at 0.15 m/s from vents and particles are injected from the mouth with a velocity of 0.15 m/s. The diameter of the virus is 0.001 25 mm.

**TABLE I.** Dimensions and geometrical parameters.

Parameter	Value (m)
*L*	6.30
*W*	5.80
*H*	3.00
*S*	0.90
*P*	0.50
*M*	2.00
*N*	0.90
*a*	0.60
*b*	0.40
*c*	2.30
*d*	1.50

### B. Governing equations

The mass and momentum conservation equations for an incompressible, turbulent, and steady-state flow can be written as follows:^28^

$$\frac{\partial \left(\rho \bar{u_{j}}\right)}{\partial x_{j}}=0,$$ (1)

$$\frac{\partial \left(\rho \bar{u_{j}}\bar{u_{i}}\right)}{\partial x_{j}}=-\frac{\partial P}{\partial x_{i}}+\frac{\partial }{\partial x_{j}}\left(\left(\mu_{f}+\mu_{t}\right)\left\{\frac{\partial \bar{u_{j}}}{\partial x_{i}}+\frac{\partial \bar{u_{i}}}{\partial x_{j}}\right\}\right)+S_{\bar{u_{i}}},$$ (2)

$$\frac{\partial \left(\rho C_{p}\bar{u_{j}}\bar{T}\right)}{\partial x_{j}}=\frac{\partial }{\partial x_{j}}\left(\left(k_{f}+\frac{\mu_{t}}{{\mathrm{Pr}}_{t}\;}\right)\frac{\partial \bar{T}}{\partial x_{j}}\right)+S_{\bar{T}},$$ (3)

where *ρ* is the fluid density, *u* is the fluid velocity, *x_i_* is the position, *t* is the time, *p* is the pressure, and *μ* is the viscosity. Since an accurate flow modeling requires a valid turbulence model, the standard k-epsilon model is utilized in this study since it has been proven to be quite effective in ventilation or low-strained flow fields. The *k-epsilon* equations are given as follows:^29^

$$\frac{\partial \left(\rho \bar{u_{j}}k\right)}{\partial x_{j}}=\frac{\partial }{\partial x_{j}}\left(\left(\mu +\frac{\mu_{t}}{\sigma_{k}}\right)\frac{\partial k}{\partial x_{j}}\right)+G_{k}-\rho \varepsilon +S_{k},$$ (4)

$$\frac{\partial \left(\rho \bar{u_{j}}\varepsilon \right)}{\partial x_{j}}=\frac{\partial }{\partial x_{j}}\left(\left(\mu +\frac{\mu_{t}}{\sigma_{\varepsilon }}\right)\frac{\partial \varepsilon }{\partial x_{j}}\right)+\frac{\varepsilon }{k}\left({C_{\varepsilon 1}p}_{k}-C_{\varepsilon 2}\rho \frac{\varepsilon }{k}\right)+S_{\varepsilon },$$ (5)

$$\mu_{t}=\rho C_{\mu }\frac{k^{2}}{\varepsilon },$$ (6)

where *μ*_t_ is the eddy viscosity; *G_k_* is the turbulent kinetic energy generation term; *σ_k_* is the kinetic energy Prandtl number; *σ_ε_* is the dissipation Prandtl number; and *C_m_*, *C_1ε_*, *C_2ε_* are the model constants. Next, the movement of the Lagrangian particles is calculated by Newton's kinematic equation as follows:

$$m\frac{du}{dt}=\sum F_{c}=F_{\mathrm{drag}}+F_{\mathrm{saff}}+F_{pg}+F_{\mathrm{grav}},$$ (7)

where *F_drag_*, *F_saff_*, *F_pg_*, and *F_grav_* are the drag force, Saffman's lift force, pressure gradient force, gravitational force, virtual mass force, basset force, and buoyancy force, respectively. Due to the small size of the particles, the drag force has the highest influence among all the forces applied on particles by the flow.^30^ The drag force can be calculated as follows:

$$F_{\mathrm{drag}}=C_{D}\rho_{f}\frac{\pi d_{P}^{2}}{8}\left|u-u_{p}\right|\left(u-u_{p}\right),$$ (8)

where *C_D_* is the drag coefficient for spherical particles calculated by using the correlations developed by Schiller–Newman over several ranges of particle *Re*. Also, the Saffman's lift force can be obtained as follows:^31^

$$F_{\mathrm{saff}}=\frac{2Ku^{\frac{1}{2}}\rho S_{ij}}{\rho d_{p}{\left(S_{ij}S_{ij}\right)}^{\frac{1}{4}}}(u-u_{p}),$$ (9)

where *K = *2.594 and *S_ij_* is the deformation tensor according to Li and Ahmadi^32^ Furthermore, the pressure gradient force *F_pg_* is defined as follows:^33^

$$F_{pg}=\rho_{f}\frac{\pi d_{P}^{3}}{6}u_{p}\nabla u.$$ (10)

Finally, the gravitational force is calculated based on the gravitational acceleration (*g*), as follows:

$$F_{\mathrm{grav}}=\rho_{p}\frac{\pi d_{P}^{3}}{6}g.$$ (11)

For the particle boundary conditions, the reflection condition was applied so the particles would remain active inside the domain and not just trapped by the walls as soon as they have been released. By doing so, the worst-case scenario would be considered for these particles.

The optimization process requires more explanation. First, various test cases must be executed, and the results must be analyzed prior to the optimization process.^34^ Second, the selected parameters used for optimization must be independent of each other,^35^ otherwise their effects cannot be studied separately. Third, it is recommended that the design points are not directly specified, and to better scatter the design points, rigorous statistical procedures such as central composite design (CCD) or Taguchi must be employed.^36^ This is important since the DOE framework involves expanding a finite number of experiments to considerable numbers. Even though the user can select his test experiments, the considered conditions might not be qualified to represent all the possible aspects of the flow. Thus, the overall sensitivity would not be reliable.^37^

In this study, the CCD method is utilized since it is considered more complex with superior performance compared to the other methods such as Taguchi or Box–Behnken design (BBD). The parameters utilized in the DOE are defined and presented in Table II.

**TABLE II.** DOE (Design of Experiment) parameters used for optimization.

	Factor 1	Factor 2	Factor 3	Response 1	Response 2	Response 3	Response 4
Run	A: Width	B: Velocity	C: Temperature	PMV	PPD	Residence time	ACE
1	0.048 344	0.221 619	294.163	−0.700 409	18.846 9	8.995	0.736
2	0.124 156	0.221 619	294.163	−1.175 08	36.583	26	0.937
3	0.048 344	0.578 381	294.163	−1.709 47	56.922 2	44	0.71
4	0.124 156	0.578 381	294.163	−2.633 48	86.3	108	0.838
5	0.048 344	0.221 619	297.137	−0.146 91	7.298 36	8.97	0.728
6	0.124 156	0.221 619	297.137	−0.501 325	13.297 8	26	0.941
7	0.048 344	0.578 381	297.137	−0.832 012	23.928 7	3.27	0.728
8	0.124 156	0.578 381	297.137	−1.462 77	48.105 1	111	0.837
9	0.0225	0.4	295.65	−0.582 262	15.206	69	0.721
10	0.15	0.4	295.65	−1.709 49	58.488 2	235	0.785
11	0.086 25	0.1	295.65	−0.123 819	6.925 85	7.77	0.886
12	0.086 25	0.7	295.65	−2.014 95	68.009	176	0.731
13	0.086 25	0.4	293.15	−1.962 88	66.389	24	0.93
14	0.086 25	0.4	298.15	−0.513 259	14.022 9	24	0.8
15	0.086 25	0.4	295.65	−1.238 34	38.797	24	0.738
16	0.086 25	0.4	295.65	−1.238 34	38.797	24	0.738
17	0.086 25	0.4	295.65	−1.238 34	38.797	24	0.738
18	0.086 25	0.4	295.65	−1.238 34	38.797	24	0.738
19	0.086 25	0.4	295.65	−1.238 34	38.797	24	0.738
20	0.086 25	0.4	295.65	−1.238 34	38.797	24	0.738

## III. RESULTS AND DISCUSSION

**FIG. 2.**
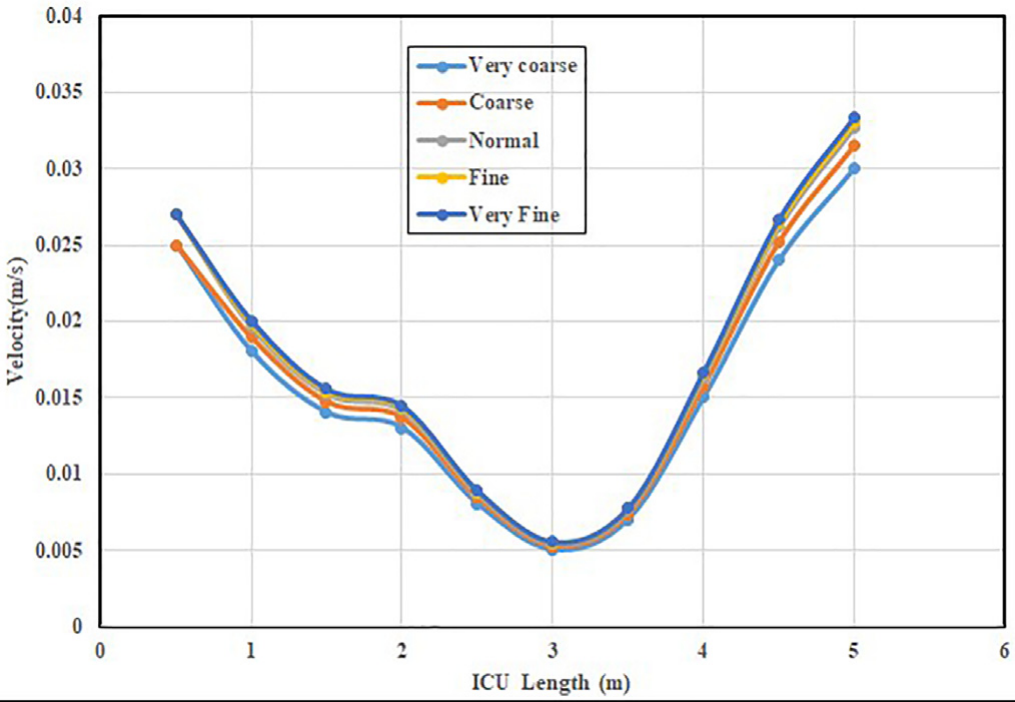
Comparison between five different mesh grids.

Prior to proceeding with a comparative study, the independence of results with regard to the grid size should be verified. For this purpose, five different mesh grids with elements between 2.5 × 10^6^ and 9.1 × 10^6^ are tested with an air inlet velocity of 0.4 m/s and temperature of 293 K and the velocity profiles along the length of the room are plotted. It can be seen from Fig. 2 that increasing the number of elements beyond the grid with 6.6 × 10^6^ elements (also known as normal) does not affect the velocity profile and, hence, this grid is used for the rest of the computation since the results are independent of the grid size.

Another important step in using any numerical scheme is checking its validity; for this purpose, an experimental study of contamination spread inside an isolation room in a hospital—which was performed by Cho^10^—is simulated and the results are compared to both the experimental and numerical data in that study. The author used sulfur hexafluoride (SF_6_) as the tracer gas in a steady-state air flow inside a 4 × 4 × 2.6 m room. SF_6_ gas was injected with an exhalation velocity of 0.955 m/s, mass fraction of 0.04, and temperature of 30 °C from a source located 0.9 m above the floor. The tracer concentrations are then measured at three different locations and compared with the results obtained from CFD. A comparison between the reported data and the results obtained in this study are shown in Table III for validation purposes.

**TABLE III.** Comparison between measured SF_6_ concentrations (ppm) from Ref. 10 and current study.

Sample point	Experiment^10^	Simulation
1	21.5	22.78
2	17.8	17.01
3	14.4	13.21

SF_6_ concentrations (ppm) are measured and reported Ref. 10 at three different points 1.4 m from the ground and are shown in Table III along with the numerical results obtained in this study. The average discrepancy between the experimental results and numerical modeling is about 6.5% for all three cases, which establishes the viability of using numerical schemes for further investigations in the current study.

### A. Flow conditions

**FIG. 3.**
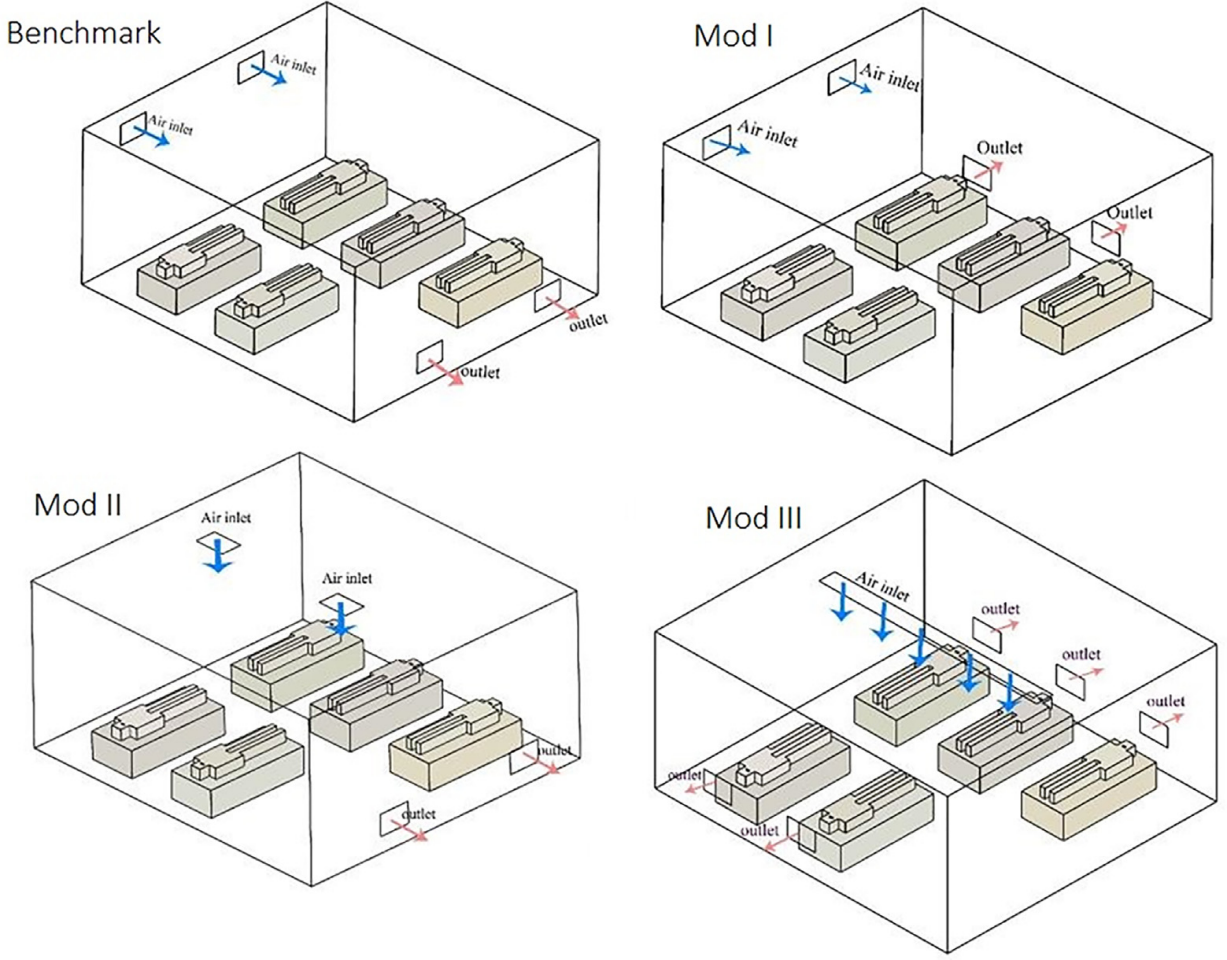
Geometry of the four different cases in the current study.

Since the viability of the grid and the numerical model used in the current study has been established and a level of confidence in the predictive capability of both is provided, the dispersion of airborne particles in an ICU room with five beds is simulated for four different ventilation systems, i.e., the benchmark and three different modifications (Fig. 3) and upon determining the case with the least spread, the parameters affecting infection control are studied. Subsequently, to further enhance the performance of ventilation systems with respect to conventional comfort metrics, an optimization process is carried out.

As mentioned before, the geometry of cases in the current study with the exception of ventilation vent locations is obtained from the work of Sahu *et al.*^7^ and, thus, for comparison the first modeled case has the exact geometry in that study and is considered the benchmark case. Two rectangular inlets and two rectangular outlets are placed in opposite walls as shown in Fig. 3. The location of vents is changed afterward: In the second case, two outlets are moved to the wall next to the two inlets, which remain unchanged. In the third case, two inlets are moved to the ceiling and the locations of the two outlets are same as the benchmark case (case I). Finally, in the last case, air is coming from one larger aspect ratio rectangular inlet in the ceiling and five outlet vents are placed on opposite side walls behind each bed.

**FIG. 4.**
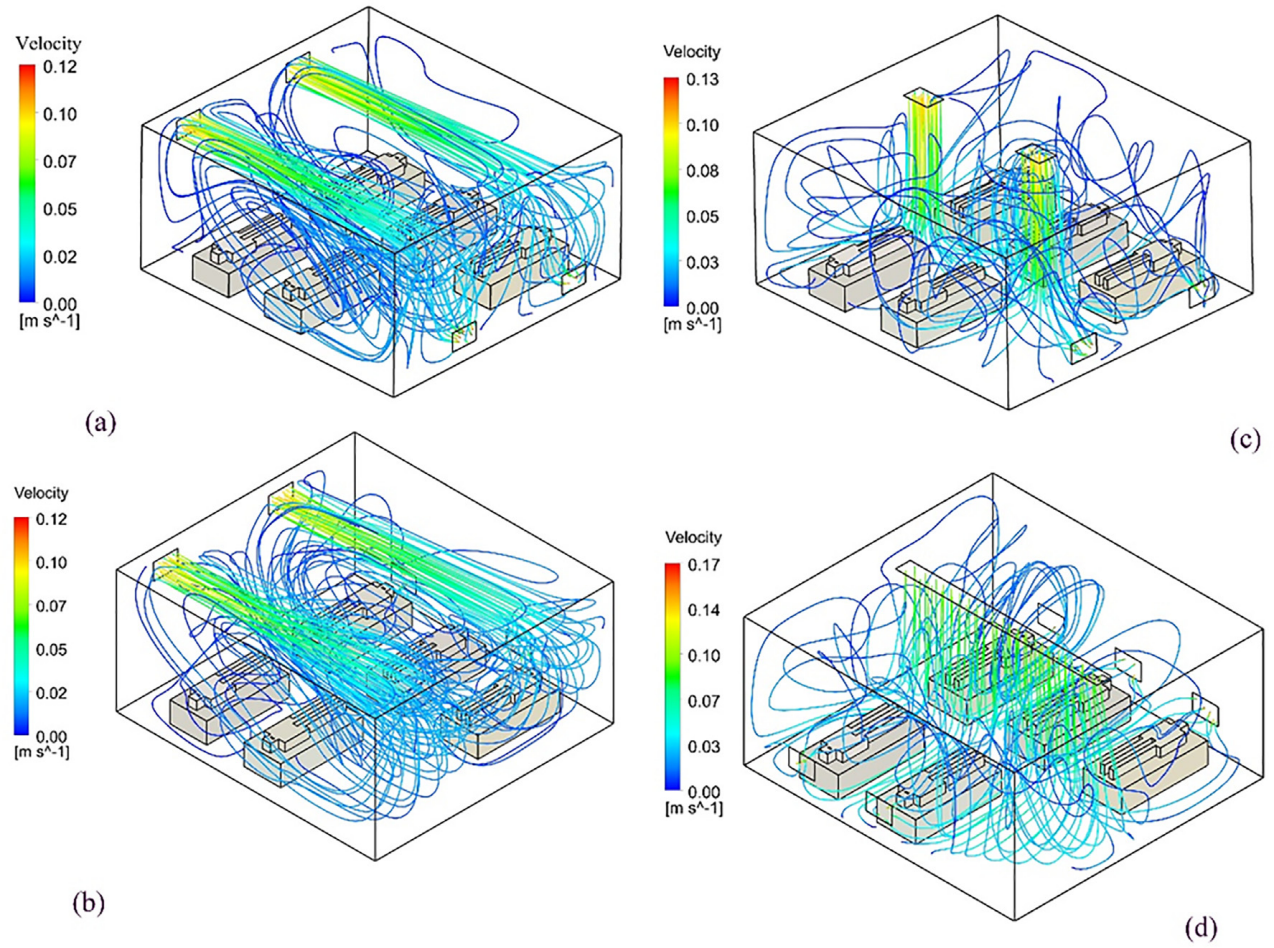
Steady-state airflow pathlines for (a) the benchmark case, (b) mod I, (c) mod II, and (d) mod III.

In all the cases, a steady-state airflow inside the room is established prior to particle injections. The pathlines of airflow for each case are shown in Fig. 4.

**FIG. 5.**
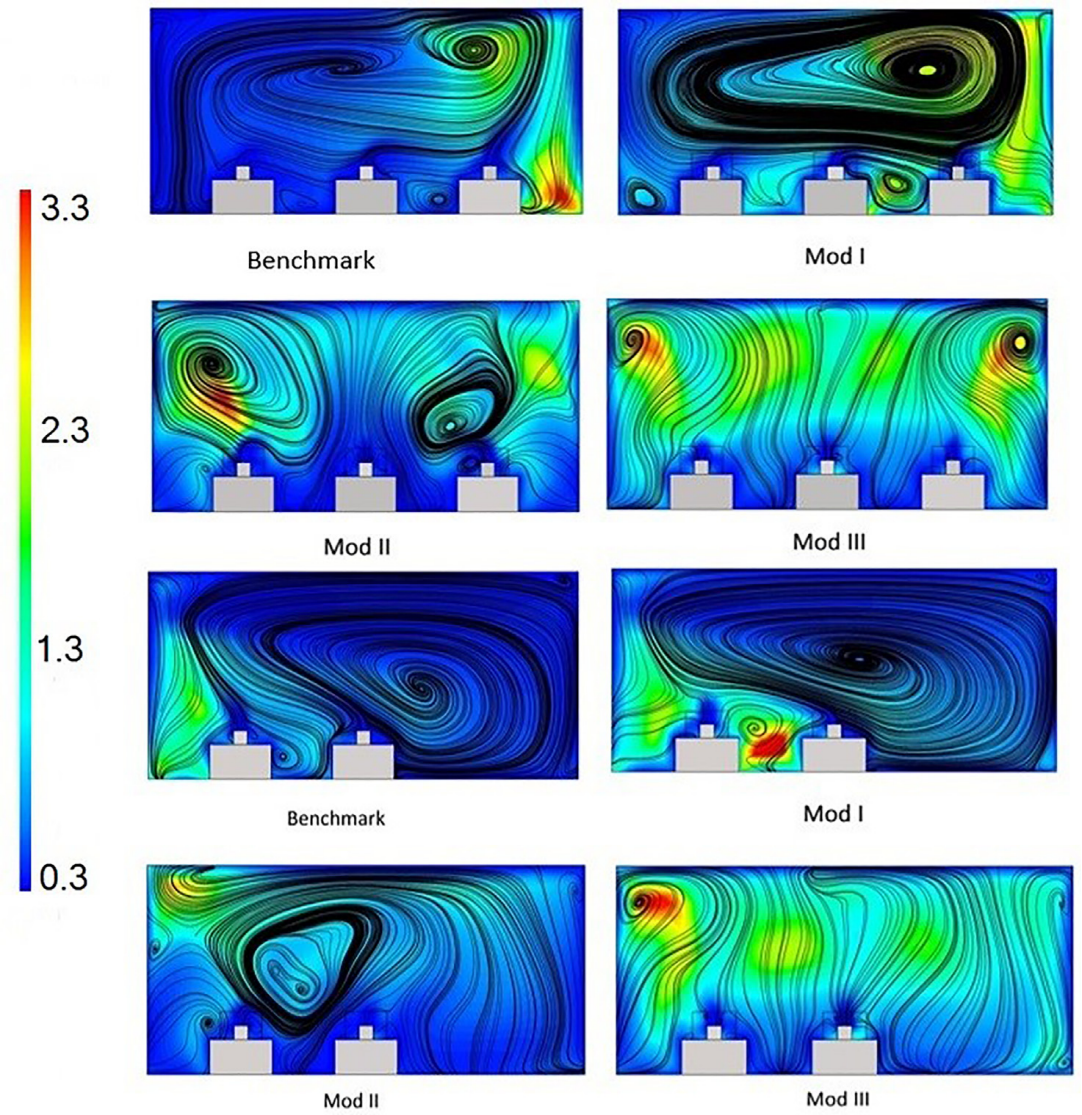
Contours of turbulent length scale and streamlines in the breathing planes of patients.

It can be seen from the flow pathlines that the ventilation system introduces a bulk flow with noticable eddy size insdie the room. To quantify the size of the largest eddies produced by the HVAC system, the turbulent length scale is calculated according to Ref. 26 as follows:

$$L_{I}=0.164{(u'}^{3}/\varepsilon ),$$ (12)

in which u' is the turbluence intensity and ε is the turbluence dissipation rate. Contours of the turbulence length scale along with streamlines are plotted in the planes of breathing for both sides of the rooms in Fig. 5.

The significance of the size of the largest eddies in the room manifests itself in the “entrapment” of airborne particles, which in turn leads to increased local particle residence time and consequently higher chance of transmission. As shown in Fig. 5, the integral length scale, which is on the order of the largest eddy, has a maximum of roughly 3 m for all cases; however, the largest eddy is furthest away from patient beds in mod III whereas in the other cases, they are located in the proximity of patients. The location and size of eddies smaller than the integral scale are also important; as can be seen from streamlines, in all cases except mod III, there are significant vortices in the bulk-flow in the domain, which essentially shows how particles will travel from one patient inside the room.

### B. Particle injections

**FIG. 6.**
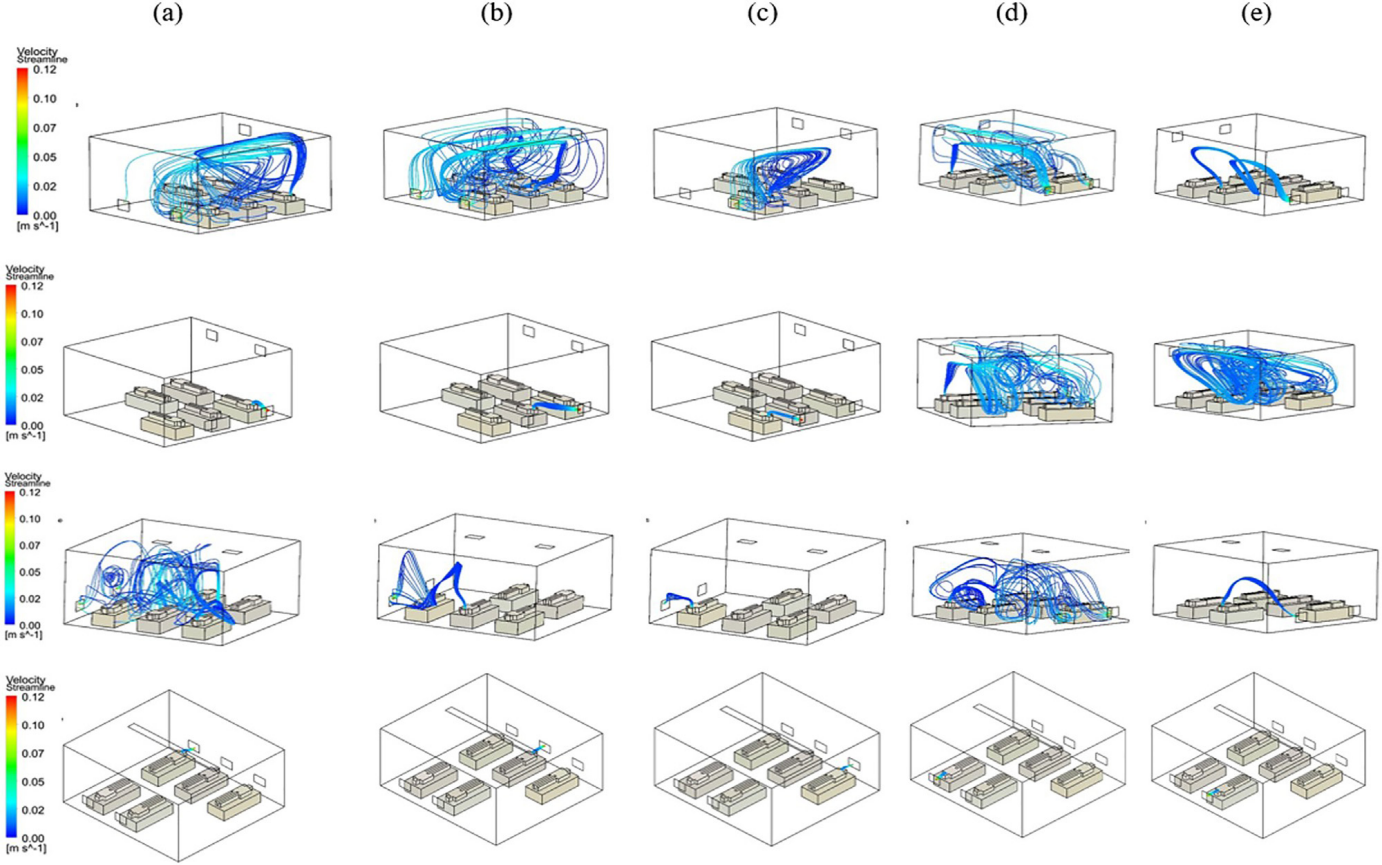
Particle pathways for patients in the benchmark case (first row), mod I (second row), mod II (third row), and mod III (forth row). Figures (a)–(e) correspond to injections from the first, second, third, fourth, and fifth bed.

To better understand the evolution of particle pathways in time, injections are done for each patient separately for all cases. Figure 6 shows these injections for all the cases. It is evident that the released particles experience a completely different pathway for each patient for a given airflow despite having the same injection mass flow rate. For instance, the maximum and minimum pathway lengths for particles from injection to the outlet vent in the benchmark case are 6.35 m for patient b and 2.13 for patient c, respectively. For case IV, these numbers are 0.9 m and 0.95 m respectively. Longer pathways correspond to larger eddy sizes which in turn means further traveling distance of particles trapped in the eddy prior to the exit from the room.

**FIG. 7.**
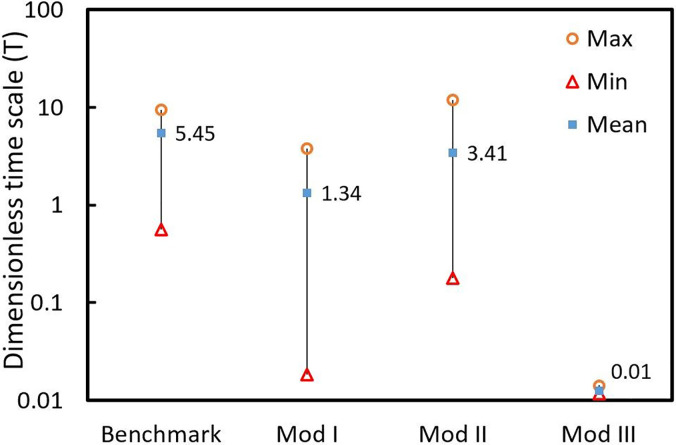
Maximum, minimum, and mean dimensionless time scales for particles averaged for all patients in each case.

Although it is evident from Figs. 5 and 6 that the ventilation system design affects infection spread significantly, the results should be quantified for accurate comparison. For this purpose, various parameters are defined for particles as well as the ventilation system. The first parameter is the dimensionless timescale, T, which is defined as

$$Τ=\frac{\tau_{\mathrm{particle}}}{\tau_{\mathrm{room}}},$$ (13)

where $\tau_{\mathrm{particle}}$ is the particle residence time (s) and $\tau_{\mathrm{room}}$ is the bulk-flow residence time (s) inside the room (which is the ratio of the room volume to the total volume flow rate, i.e., $\tau_{\mathrm{room}}=\frac{V}{\dot{V}}$ = 548.1 s). Three values of the minimum, maximum, and mean dimensionless timescale of particles averaged for all patients in each case are calculated and plotted in Fig. 7.

The larger the value of T, the longer it takes for the particles to exit the room and consequently the higher the chance of airborne exposure for the staff and non-infected patients. It is evident from Fig. 7 that a poorly designed ventilation system, the benchmark case for instance, can prolong the particle residence time by an order of magnitude. A smaller value of T, in contrast, minimizes the risk of contamination spread. Mod III, for example, has a mean T of 0.01, meaning that the average time a particle spends in the room is less than 6 s [compared to ∼6000 (s) in the benchmark case]. Another point worth noting is the large residence time distribution, which shows the degree to which contamination spread is dependent on the location. Ideally, there should not be any correlation between the location of infected patients and the level of spread of the infectious agent and this occurs only in mod III since the difference between the maximum and minimum T is negligible. For other cases, however, there is at least one order of magnitude difference in T, which implies a high level of dependency of spread to location.

Another parameter is the extracted mass of injected particles (m_e_) normalized by the total injection mass (M), i.e.,

$$m_{n}=\frac{m_{e}}{M}.$$ (14)

**FIG. 8.**
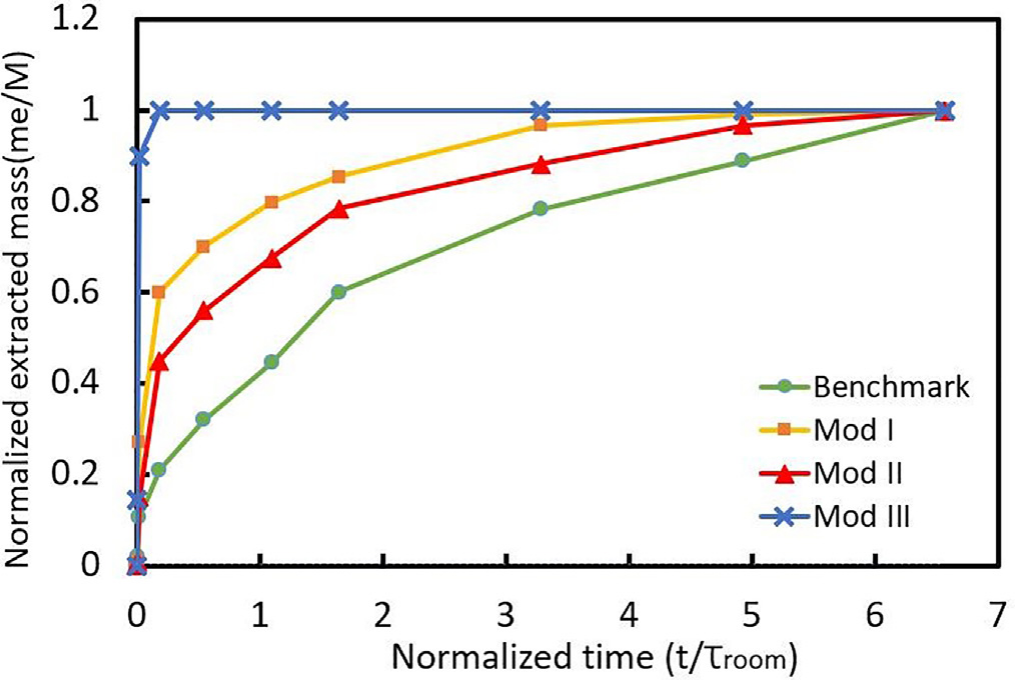
Profiles of normalized extracted mass of particles with respect to dimensionless time (t normalized with bulk flow residence time $\tau_{\mathrm{room}}$).

This normalized extracted mass of particles (m_n_) is 0 at the time of injection and will be 1 when the mass is completely extracted from room. m_n_ is measured over time for each patient and then averaged for each case and the results are plotted in Fig. 8.

It can be seen from Fig. 8 that m_n_ for all cases goes to 1 ultimately, which means that all the injected mass exits the room eventually. However, the time over which maximum extraction occurs determines the effectiveness of the ventilation system. For the benchmark case, it takes about 6.7 times the bulk-flow residence time (3600 s) for the ventilation system to empty the room from particles. This would be done in less than 10 s (less than 0.02 of $\tau_{\mathrm{room}}$) in mod III when the air enters from the ceiling and exits from both sides of the room. Figure 8 shows that mod I, in which the air comes from the ceiling again, performs better than the benchmark case and mod II, but is still not as good as mod III. Results from Fig. 8 could be used to define another timescale associated with the extraction of injected material for each ventilation system. By applying a simple mass balance, *In + Generation − Out = Accumulation*, on the particles after injection, we have

$$0+0-c{}_{\mathrm{out}}\dot{v}=V\frac{dc}{dt},$$ (15)

in which *c* is the concentration, $\dot{v}$ is the volumetric flow rate, and *V* is the total volume of the room. Equation (7) is an ordinary differential equation, which can be solved through separation of variables and integration as follows:

$$-\dot{\frac{v}{V}}dt=\frac{c}{c{}_{\mathrm{out}}}\to \;\int_{c_{0}}^{c_{\mathrm{out}}}\frac{dc}{dc_{\mathrm{out}}}=-\int_{0}^{t}\frac{\dot{v}}{V}dt\to \ln\left(\frac{c_{\mathrm{out}}}{c_{0}}\right)=-\frac{t}{\tilde{\tau }}.$$ (16)

Therefore, if the logarithm of the effluent particle concentration normalized by the initial concentration (ln(c/c_out_)) is plotted with respect to time, the extraction timescale ( $\tilde{\tau }$) is easily obtained by taking the slope of the plot,

$$\tilde{\tau }=-\frac{1}{\frac{d}{dt}\left(\ln\left(\frac{c_{\mathrm{out}}}{c_{0}}\right)\right)}.$$ (17)

**FIG. 9.**
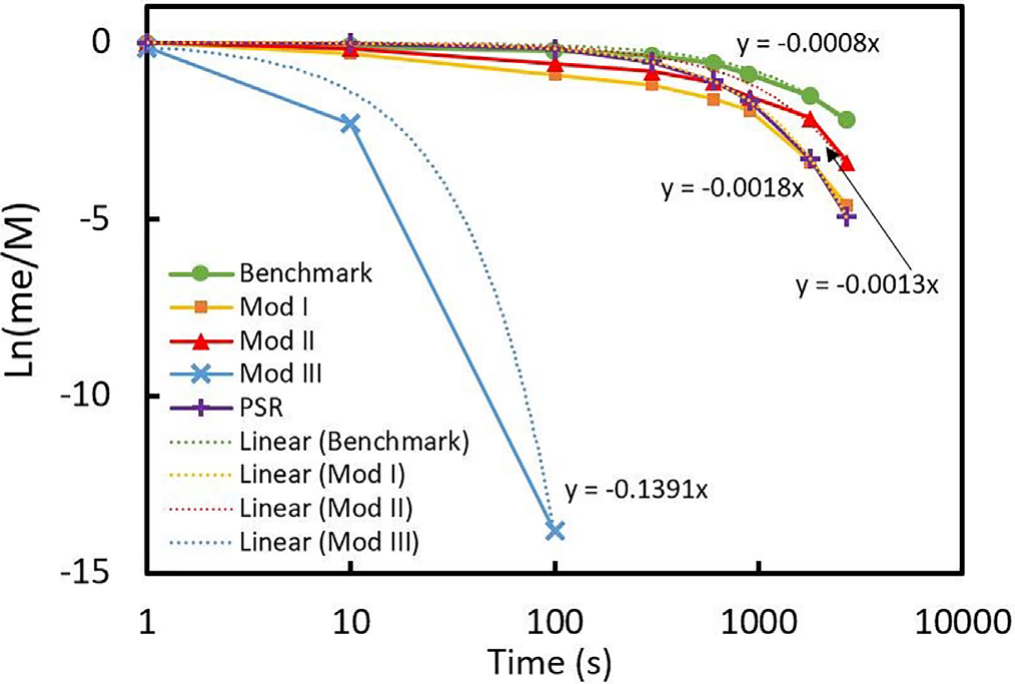
Profiles of normalized effluent particles as a function of time for all cases averaged for all patients.

To find this time for each case, graphs of Ln(m_e_/M) averaged for all patients are plotted and the slopes are calculated using linear fitting as shown in Fig. 9. It should be noted that when $\tilde{\tau }$ = $\tau_{\mathrm{room}}$, Eq. (16) shows the performance of a perfectly stirred reactor (PSR); so for comparison, the PSR predictions are also plotted.

It is evident from Fig. 9 that the extraction time for each case is −1/(*dy*/*dx*) of the fitted line. Therefore, $\tilde{\tau }$ is 1250, 769.2, 555.5, and 7.2 s for the benchmark (case 1), mod II, mod I, and mod III respectively. This further proves that the ventilation system in mod III outperforms all the other cases as it can completely extract the particles up to four orders of magnitude faster than the other cases. Hence, this is proposed as the most effective ventilation system to control infection spread inside the patient rooms. By comparing the results with the PSR predictions, it can be concluded that except mod III, all modeled cases act similar to a PSR, which as suggested by its name and by definition guarantees perfect homogeneity of the mixture inside a control volume, which can be catastrophic when it comes to mixing of a contagious agent such as SARS-CoV-2 virus in a room. In fact, in places like ICUs or isolation rooms, the mixing characteristic of air conditioning systems should be far from perfect and the only case in which this is achieved is mod III.

### C. Effects of geometry and optimization

After it is stablished that in the considered scenarios, mod III—in which air is coming from the ceiling and the outlet vents are placed on both walls behind the patient beds—performs the best, the effects of three different parameters, viz., inlet channel width (W), inlet air velocity (V), and inlet air temperature (T), on the system performance are studied in a systematic way and an optimization scheme is carried out to enhance the performance even further. The optimization scheme of the RSM is used and 20 design points are considered using the central composite design method, and three different response surfaces are created: 1—*predicted mean vote* (PMV), 2—*percentage of people dissatisfied* (PPD), and 3—*air change effectiveness* (ACE). These are standard parameters introduced by ASHRAE and are used to determine the thermal comfort of ventilation systems. PMV is calculated as follows:

$$\mathrm{PMV}=3.155\left(0.303e^{-0.114M}+0.028\right)L,$$ (18)

where *M* is the rate of metabolic generation and *L* is the thermal load, which is calculated based on numerous parameters such as metabolic heat loss, surface temperature of the body, and effective thermal resistance of clothing material.^27^

The PPD is then calculated using PMV from Ref. 27 as follows:

$$\mathrm{PPD}=100-95\;\exp\left(-0.033\;53\;\mathrm{PM}V^{4}-0.2179\;\mathrm{PM}V^{2}\right).$$

**FIG. 10.**
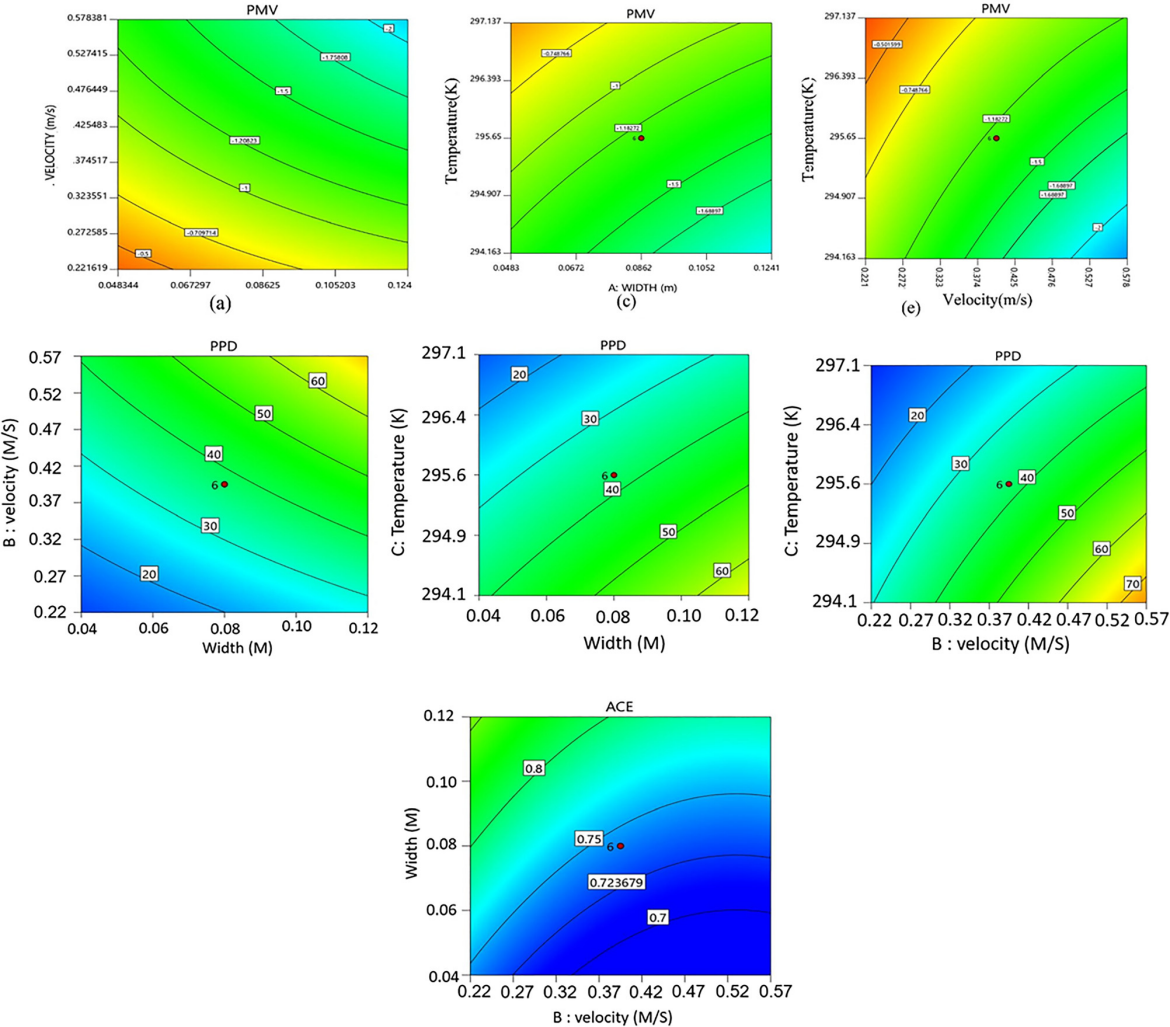
Surface responses of PMV and PPD to studied variables; effects of velocity and channel width (left column), temperature, and length (middle column), and temperature and velocity (right column). Surface response of ACE (last row) to channel width and air velocity at T = 297.13 K.

For the optimization process, the width is varied from 0.048 to 0.15 m, the velocity is varied from 0.22 to 0.58 m/s, and temperature is varied from 294.1 to 297.1 K. Contour maps of PMV and PPD are shown in Fig. 10.

Effects of varying the mentioned parameters on comfort conditions are visible in Fig. 10. According to ASHRAE, a PMV of zero represents thermal neutrality and changes between −0.5 and 0.5 are considered within the comfort zone. The comfort zone would normally have a PPD of less than 20%, which means the occupant satisfaction rate is more than 80% for a given thermal condition.^27^ For a constant channel width, as the velocity is increased the PMV is decreased to larger negative values, i.e., less comfortable conditions. Likewise, as the velocity is increased for a constant width, the PPD increases. It should be noted even for small values of velocity, PMV and PPD exit the comfort zone conditions as the width is increased beyond certain values. Increasing the temperature for a constant channel width on the other hand increases the PMV from larger negative numbers, for example, −2 at T = 294.16 K toward the confront zone limit, −0.5 at 297.14 K. Similarly, the PPD gets closer to the comfort conditions as the temperature is increased at a constant width. Finally, both PMV and PPD approach the comfort limit as the velocity is decreased at constant temperature. It is worth noting that the iso-values of PMV and PPD in Fig. 10 show that for all three parameters, a certain value can be obtained through various choices of velocity, channel width, and temperature. In other words, there is a range of parameters in which thermal conditions are considered desirable.

The ACE is a measure of air displacement within an enclosed environment. It is the ratio of air residence time to the average age of air at a reference height (typically breathing height); according to ASHRAE criteria for modern buildings, it should be larger than 0.95. An ACE of unity represents perfect mixing of the air distribution; hence, to avoid local stagnation of air ACE should be close to unity across the room. Since ACE is defined based on the ventilation system's residence time, it can be rewritten using T (dimensionless timescale) in Eq. (13) as follows:

$$\mathrm{ACE}=\frac{\tau_{\mathrm{room}}}{\tau_{\mathrm{avg}}}=\frac{1}{T}\frac{\tau_{\mathrm{particle}}}{\tau_{\mathrm{avg}}}.$$ (19)

Here, $\tau_{\mathrm{avg}}$ is the average age of air and is calculated utilizing a user-defined scalar inside the FLUENT software.

Like PMV and PPD, for a constant width, as the velocity decreases the ACE approaches the comfort condition. Using the contours in Fig. 10, three different quadratic fittings are proposed for calculating PMV, PPD, and ACE as a function of width, temperature, and velocity as follows:

$$\text{PMV}=13.388\;-277.915W-117.725V\;+\;0.0475T\;-13.411WV+0.916WT+0.386VT+22.832W^{2}+\;1.880V^{2},$$ (20)

PPD=−165.498+11172.9W+5117.4V+0.532T+551.113WV−37.556 WT−17.133VT, (21)

ACE=1712.06+1.465W−0.784V−11.566T+0.729V2. (22)

Using the above equations, we eliminate all the other variables that may not be readily available during the design process and make the calculations easy.

**FIG. 11.**
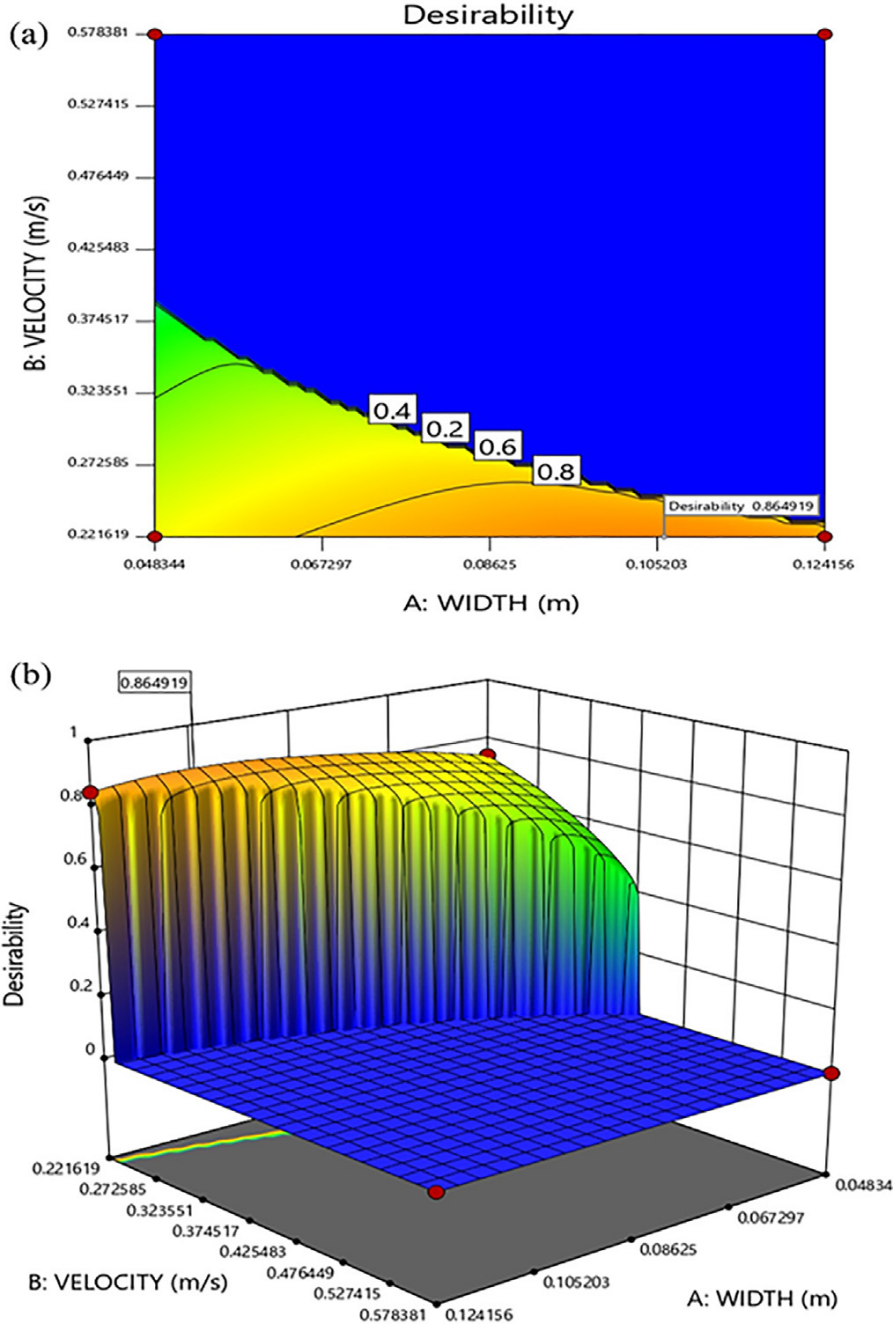
Contours of desirability as a function of width and velocity at T = 297.13 K for a 2D cross section of velocity and width (a) and full 3D response surface (b).

As mentioned before, the response surfaces in Fig. 10 show that thermal comfort condition is obtained within a range of values for the three studied parameters, hence a desirability function is defined based on PMV, PPD, and ACE criterion that shows the limits for variable selection. The ideal thermal conditions correspond to the desirability of 1, and by plotting the response surfaces one can easily select the parameters that provide comfortable conditions. The desirability surfaces are plotted in Fig. 11.

**FIG. 12.**
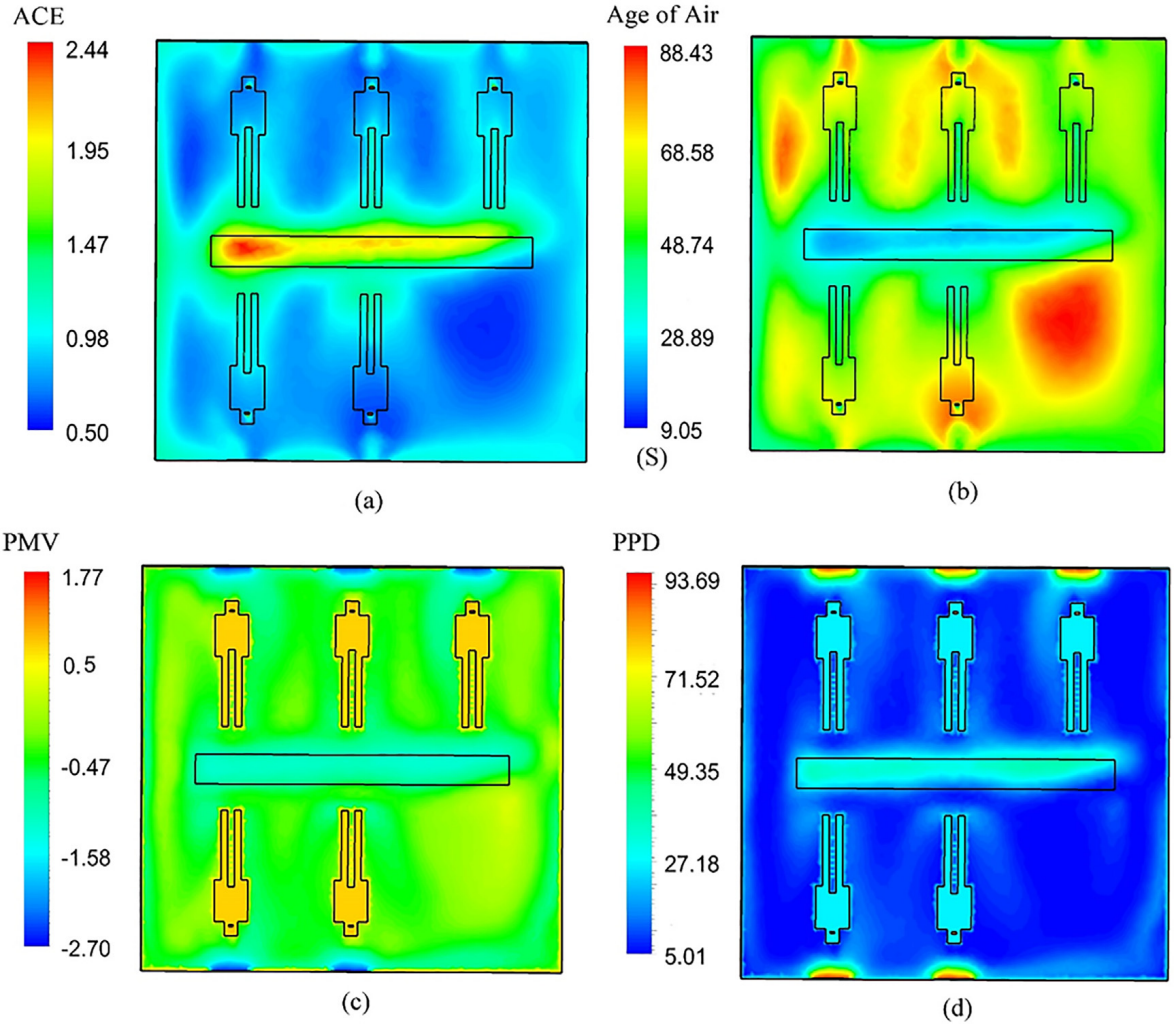
Contours of thermal comfort parameters for a fixed channel width, air velocity, and air temperature: (a) ACE, (b) age of air, (c) PMV, and (d) PPD.

The maximum desirability obtained in the current study is 0.865 as shown in Fig. 11. This value can be obtained through various design selections. For example, for a velocity of 0.22 m/s and temperature of 297.137 K any value of width between 0.104 and 0.108 m results in a desirability of 0.865. To show the performance of the ventilation system and the distribution of thermal criterion, another set of computation is performed with fixed values of channel width, air inlet velocity, and temperature and the contours of PMV, PPD, ACE, and age of air are plotted in Fig. 12.

Even though the predicted values for standard thermal comfort parameters fall in the suggested range, the distribution of these parameters are also significant as the ideal condition must have a uniform distribution. Figure 12 shows that not only the predicted values for PMV, PPD, and ACE are close to ideal conditions, the distribution of parameters specifically in proximity of patient beds is in fact uniform, which further proves the ability of the proposed design to provide thermal comfort.

## IV. CONCLUSIONS

The effects of design parameters on the performance of ventilation systems to control the spread of airborne particles in an ICU are studied numerically. Four different cases with various locations of inlet and outlet vents are considered and the spread of particles is studied. Two new criteria for the ventilation system are defined—viz., dimensionless timescale (T) and extraction timescale $\tilde{\tau }$—and the performance of all the cases are compared. The results show that when the air inlets are placed in the ceiling and outlets are placed behind the patient beds (mod III), the infection spread is least probable since the particles exit the room 2 orders of magnitude faster than the other cases. Once it is established that this system provides a significant enhancement in infection control, an optimization process is performed to understand the effects of design variables (air inlet width, inlet air velocity, and inlet air temperature) on the thermal comfort conditions (PMV, PPD, ACE) according to the suggested standard values, and the relations for calculating each of these parameters based on the design variables are proposed. Desirability functions that are comprised of all three thermal condition parameters are used to determine the range of variables that result in thermally comfortable conditions and a maximum desirability of 0.865 is obtained.

## Data Availability

The data that support the findings of this study are available from the corresponding author upon reasonable request.
